# Detection of Total Aflatoxins in Herbal Medicines Based on Lateral Flow Assay with Contamination Ratio Model

**DOI:** 10.3390/molecules29245827

**Published:** 2024-12-10

**Authors:** Xiao-Ya Qin, Rui Feng, Heng Zhou, Hui-Qin Pan, Hao Wang, Xiao-Jing Huang, Jian-Ying Shen, Qing Hu, Shen Ji

**Affiliations:** 1 School of Pharmacy, Shanghai University of Traditional Chinese Medicine, 1200 Cailun Road, Shanghai 201203, China; qinxiaoya0908@163.com (X.-Y.Q.); 15084940082@163.com (H.W.); 2 NMPA Key Laboratory for Quality Control of Traditional Chinese Medicine, Shanghai Institute for Food and Drug Control, 1500 Zhangheng Road, Shanghai 201203, China; fengrui-vc@163.com (R.F.); phq20095399@163.com (H.-Q.P.); hxj0201@126.com (X.-J.H.); 13311603693@163.com (J.-Y.S.); huqingyjs@163.com (Q.H.)

**Keywords:** colloidal gold immunochromatographic strip, aflatoxins, herbal medicines, rapid quantitation detection, matrix effect

## Abstract

In this study, we developed a colloidal gold immunochromatographic strip (CGIS) method that used the matrix-matched calibration curves of contamination ratio models to quantitatively determine the total aflatoxin in five herbal medicines. This approach addresses issues related to false results and poor accuracy associated with conventional methods. The CGIS was analyzed using a Vertu touch reader, and the matrix-matched calibration was established based on the absorbance ratios of the T and C lines, as well as the logarithmic values of the total aflatoxin concentrations. The total aflatoxins could be accurately and digitally detected from 2.5 to 40 μg/kg, and the LOD of total aflatoxins was 1 μg/kg in the five herbal medicines. The recovery rates from the spiked samples ranged from 65.1% to 98.6%, and the RSD was less than 16.9%. A total of 229 samples were analyzed by both CGIS and HPLC-FLD, with agreement ranging from 78.4% to 132.6% (*Arecae semen*), 82.6% to 133.0% (*Nelumbinis semen*), 79.9% to 117.9% (*Coicis semen*), 78.1% to 119.0% (*Platycladi semen*), and 76.1% to 123.0% (*Ziziphi spinosae semen*). This process for the discrimination of the CGIS results was established to assess if samples met the requirement of aflatoxin limits, which could save approximately 75% in time and reduce the workload of retesting by a designated confirmatory reference method to less than 10%. This study demonstrated that the application of matrix-matched calibration curves based on contamination ratio models to CGIS can effectively enhance the rapid quantitative determination capability of total aflatoxins in herbal medicine matrices.

## 1. Introduction

There has been a notable global surge in recent decades in the popularity of herbal medicines [1,2]. The World Health Organization estimates that approximately four billion people worldwide currently use herbal medicines for therapeutic purposes [3]. This increasing use has raised significant concerns among health authorities and the public regarding mechanisms for ensuring product quality [4,5]. Aflatoxins are toxic secondary metabolites that are naturally produced by certain fungi, such as *Aspergillus parasiticus* and *A. flavus* [6,7,8]. In recent years, there have been frequent reports of aflatoxin contamination in various herbal medicines, including *C. semen*, *Ophiopogon japonicus*, *P. semen*, *Scaphium wallichii*, *A. semen*, and *Z. spinosae semen* [9,10]. More than 20 aflatoxins have been identified, with aflatoxin B_1_ (AFB_1_), aflatoxin B_2_ (AFB_2_), aflatoxin G_1_ (AFG_1_), and aflatoxin G_2_ (AFG_2_) being the most prevalent and significant [11,12]. Aflatoxins have been associated with various acute and chronic ailments in humans, including hepatic carcinoma, impaired growth, compromised immune function, pyrexia, emesis, and hepatic insufficiency [13]. In light of these adverse effects, over 75 countries have implemented strict regulatory measures to control aflatoxin levels in food and agricultural products, including herbal medicines [14]. The Chinese Pharmacopoeia (2020 edition) prescribes thresholds of 5 µg/kg for AFB_1_ and 10 µg/kg for total aflatoxins in herbal medicines [15].

Rapid screening techniques are crucial for monitoring aflatoxins through the various stages of collection, processing, transportation, and storage of herbal medicines [16,17,18]. The colloidal gold immunochromatographic strip (CGIS) method, which is one of the most commonly used lateral flow assay technologies, is now wildly applied in the on-site detection and large-scale screening of aflatoxins [19,20]. It offers several advantages, including rapid analysis speed, simple sample preparation, affordability, and portability for field use [21]. However, the CGIS method is mostly used for the qualitative or semi-quantitative detection of aflatoxins. It still faces many challenges for the quantitative detection of aflatoxins in complex herbal medicines. The Chinese Pharmacopoeia (2020 edition) and European Pharmacopoeia stipulate that total aflatoxins includes four major aflatoxin subtypes, namely, AFB_1_, AFB_2_, AFG_1_, and AFG_2_. Currently, these four aflatoxin subtypes exhibit inconsistent affinities with antibodies in total aflatoxins immunoassays [22,23]. Furthermore, the types and contamination ratios of these subtypes vary across different matrices [1,10]. Consequently, the results obtained from CGIS methods for determining total aflatoxin levels often differ significantly from those obtained using HPLC-FLD and other analytical instruments. In addition, naturally positive quality control samples from certain complex matrices are both costly and difficult to obtain, and solvent standard curves are inadequate for correcting the matrix effect. Therefore, it is essential to design and establish a stable and controllable method for preparing calibration curves that does not rely on positive quality control samples. This approach aims to address the poor level of accuracy in the rapid quantitative determination of aflatoxins using the CGIS method, which is caused by variations in the aflatoxin ratio and the matrix.

In this work, we modeled the contamination ratios of four aflatoxins (AFB_1_, AFB_2_, AFG_1_, and AFG_2_) in five herbal medicines, based on the monitoring data of naturally contaminative herbal medicines recorded in our in-house database. Then, matrix-matched calibration curves were developed for the herbal medicines *A. semen*, *N. semen*, *C. semen*, *P. semen*, and *Z. spinosae semen* by adding aflatoxin standards to the blank matrix according to the contamination ratio models of the four aflatoxins. Finally, the results obtained from CGIS were compared and analyzed against those from high-performance liquid chromatography with a fluorescence detector (HPLC-FLD) to evaluate the quantitative reliability of the CGIS across five herbal medicines.

## 2. Results and Discussion

### 2.1. Sensitivity and Specificity of the CGIS

To assess the ability of the CGIS to detect the total aflatoxins in herbal medicine matrices, it was necessary to investigate the cross-reactivity of the test. Four aflatoxins (AFB_1_, AFB_2_, AFG_1_, and AFG_2_) were applied separately to the CGIS and exhibited different cross-reactivities, with AFB_1_ and AFG_1_ displaying the strongest affinities (Figure S1). These differing reactivities would, therefore, produce different screening results for the individual aflatoxins. Thus, it was necessary to establish ratio models for each matrix based on the contaminative level of the four aflatoxins in the five herbal medicines. Furthermore, some mycotoxins (including OTA, o-m-ster, DON, ZEN, and T-2) were detected using the CGIS with concentrations of 2 μg/mL for each, and the results indicated an absence of cross-reactivity (Figure S2).

As mentioned above, the sensitivity of the strips was evaluated by subjecting them to a series of decreasing concentrations of aflatoxin standard solutions. The lowest concentration at which the strips exhibited a significant difference compared to the blank sample was identified as the LOD, and the results are shown in Figure S3. The significant differences (*p* < 0.05) were observed between strips of the blank samples of five herbal medicine matrices and strips exposed to total aflatoxins at a concentration of 1 µg/kg. The results indicated that the sensitivity of the test strip was good, meaning it could meet the requirements for the rapid quantitative determination of total aflatoxins.

### 2.2. Quantitative Difference of Calibration Curves Constructed Using Various Aflatoxin Ratio Models

**Figure 1:**
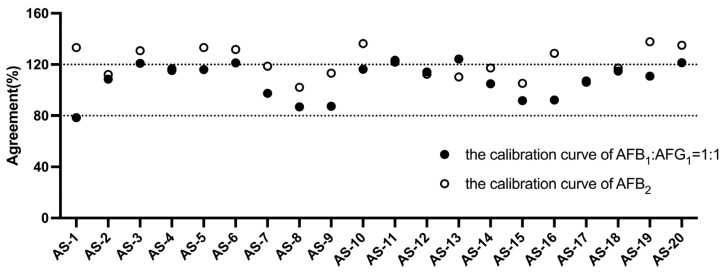
The agreements of *A. semen* samples were determined under different calibration curves.

To assess the efficacy of the ratio model, we took *A. semen* as an example and constructed two distinct calibration curves, including the calibration curve AFB_1_:AFG_1_ = 1:1 (which was constructed using the ratio model) and the calibration curve AFB_2_, to quantify the total aflatoxins from *A. semen* positive samples. We then compared the agreements among 20 batches of positive samples on the two calibration curves to evaluate the model. Figure 1 shows that the agreements (the ratio) between the total aflatoxins of the *A. semen* samples obtained using the calibration curve AFB_1_:AFG_1_ = 1:1, and those measured by HPLC-FLD, were within an acceptable range. However, the agreements (the ratio) between the CGIS results obtained using the calibration curve AFB_2_, and the HPLC-FLD results, were generally outside the acceptable range. This indicated that the CGIS calibration curves were more suitable for the accurate quantification of aflatoxins based on the contamination ratio model of four aflatoxins in five herbal medicine matrices.

### 2.3. Influence of Herbal Medicine Matrices

**Figure 2:**
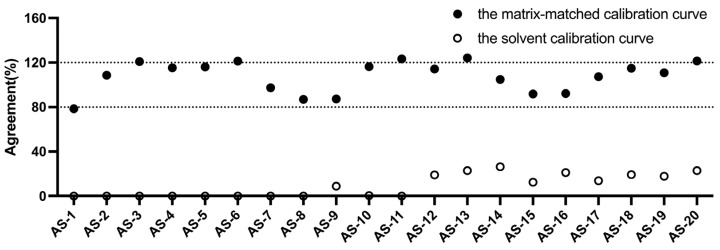
The agreements of *A. semen* samples were determined under different calibration curves.

Currently, CGIS is widely used only in maize, wheat, and single-matrix samples that are little disturbed by pigmented backgrounds [24]. In contrast, herbal medicines such as *A. semen*, *N. semen*, *C. semen*, *P. semen*, and *Z. spinosae semen* present a complex matrix due to their deep pigmentation, oils, and aromatic compounds. This intricate matrix, along with potential cross-reactions, may compromise the accuracy of aflatoxin test results and could even lead to false results [25]. To address this issue, we attempted to establish a matrix-matched calibration curve to solve the false results and poor accuracy caused by complex matrix effects. Taking *A. semen* as an example, we compared the accuracy of sample detection by establishing a solvent calibration curve and a matrix-matched calibration curve. As shown in Figure 2, the agreements of the *A. semen* samples were beyond the acceptable range under the solvent calibration curve, whereas the matrix-matched yielded agreements fell predominantly within the acceptable range, which indicated that constructing a matrix-matched calibration curve could improve the accuracy of the results. In addition, the matrix-matched calibration curve constructed by the CGIS could be added into a strip reader for subsequent detection.

### 2.4. Quantitative Detection of the CGIS and Methodology Validation

**Figure 3:**
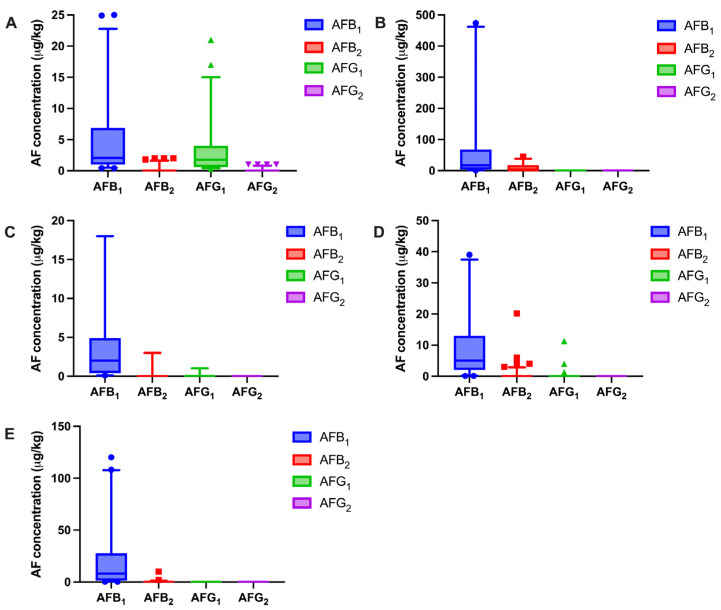
The distribution of the four types of aflatoxins in *A. semen* (**A**), *N. semen* (**B**), *C. semen* (**C**), *P. semen* (**D**), and *Z. spinosae semen* (**E**), respectively.

Prior to building a matrix-matched calibration curve, a specific contamination ratio model of AFB_1_, AFB_2_, AFG_1_, and AFG_2_ was established for each herbal medicine (*A. semen*, *N. semen*, *C. semen*, *P. semen*, and *Z. spinosae semen*). The contamination ratio was obtained by normalizing the contamination data of the aflatoxins from the in-house database (Figure 3 and Table 1). In constructing the contamination ratio model, the ratios of AFB_1_, AFB_2_, AFG_1_, and AFG_2_ in the *A. semen* were 0.88:0.02:0.76:0.001, respectively. Given the low concentrations of AFB_2_ and AFG_2_, the contamination ratios of the four aflatoxins were approximated as 1:0:1:0. This approach was similarly applicable to the other herbal medicines. For *N. semen*, the ratios of AFB_1_, AFB_2_, AFG_1_, and AFG_2_ were 0.95:0.11:0.002:0, respectively. For *P. semen*, the ratios of AFB_1_, AFB_2_, AFG_1_, and AFG_2_ were 0.94:0.07:0.006:0, respectively. For *Z. spinosae semen*, the ratios of AFB_1_, AFB_2_, AFG_1_, and AFG_2_ were 0.96:0.06:0.009:0.003, respectively. Given the low concentrations of AFG_1_ and AFG_2_, the contamination ratios of the four aflatoxins were approximated as 10:1:0:0. For *C. semen*, the ratios of AFB_1_, AFB_2_, AFG_1_, and AFG_2_ were 1:0.007:0:0, respectively, leading us to approximate the contamination ratio of the four aflatoxins as 1:0:0:0. The models obtained were then applied in validating the in-house database. The results showed that 64.0% of the *A. semen* batches, 88.9% of the *N. semen* batches, 72.2% of the *C. semen* batches, 83.3% of the *P. semen* batches, and 95.5% of the *Z. spinosae semen* batches exhibited contamination ratios that were within ±20% of the set aflatoxin ratio models.

**Figure 4:**
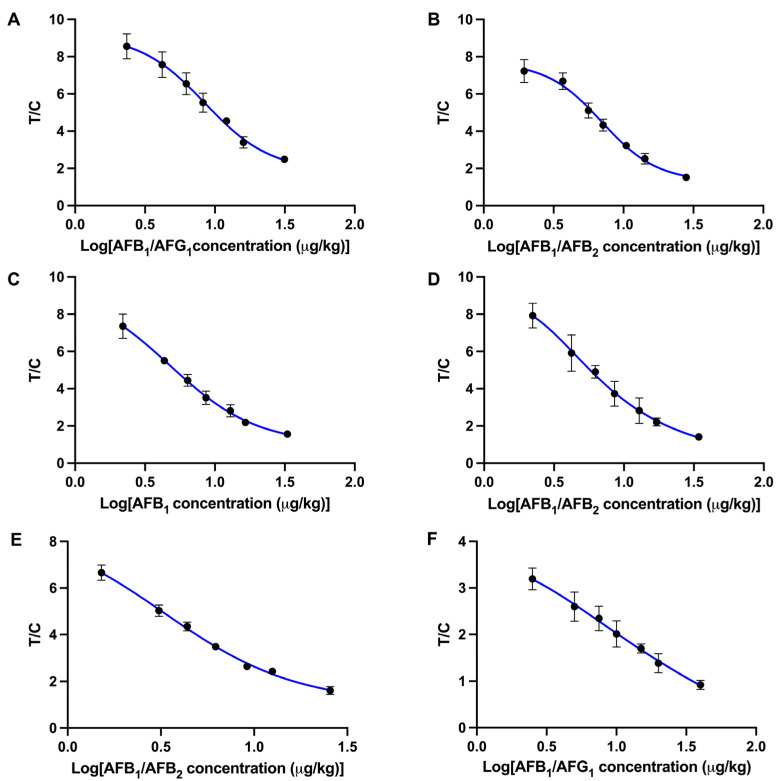
The calibration curves for aflatoxin detection. The four-parametric logistic calibration curves were created by plotting the ratio of T and C against the concentration of aflatoxins (the calibration curve concentration was obtained by HPLC-FLD detection) in *A. semen* (**A**), *N. semen* (**B**), *C. semen* (**C**), *P. semen* (**D**), *Z. spinosae semen* (**E**), and the solvent standard (**F**), respectively.

The CGIS for the determination of the total aflatoxins was developed based on a competitive immunoassay format. The analytes in the sample competed against the immobilized T line reagent binding to the gold-labeled monoclonal antibodies, thus resulting in a decrease in or disappearance of the coloration dot strip. To obtain a quantitative detection, a Vertu TOUCH reader was used to scan the CGIS, and the calibration curve of the contamination ratio for each herbal medicine was obtained. The absorbance ratios of the T and C lines (Figure S4) were plotted against the logarithmic values of the aflatoxin calibrant concentrations, which fitted a four-parameter logistic model (Figure 4). The calibration curve equations for the five herbal medicines, listed in Table S1, exhibited correlation coefficients (R^2^) above 0.95. After assessing the accuracy of the CGIS, the recoveries of the spiked samples were found to be between 65.1% and 98.6%, with the RSD of the spiked samples between 1.6% and 16.9% in the five herbal medicines (Table S2). Additionally, the reproducibility of the CGIS method was evaluated by three different analysts using *A. semen* quality control samples with a total aflatoxin concentration of 8.0 μg/kg. Twenty-one of the results fell within the acceptable ranges, and the RSD value for the data generated by the three analysts was 9.6% (Table S3). These findings demonstrate that the CGIS shows excellent precision and accuracy, so it can be used for the quantitative detection of the total aflatoxins in herbal medicines.

### 2.5. Process for Discrimination of CGIS Results

The Chinese Pharmacopoeia (2020 edition) stipulates that the maximum allowable limit for total aflatoxins in herbal medicines is 10 μg/kg, while the Design Criteria and Test Performance Specifications for Quantitative Aflatoxin Test Kits (DSTPSQATK) Accuracy Rules, established by the US Department of Agriculture’s Federal Grain Inspection Service (FGIS), state that the RSD at 10 μg/kg should not exceed 23%. The formula for this is Maximum RSD (%) = 31.572·C^−0.149^, where C is the aflatoxin concentration in μg/kg [26]. In this work, authentic samples of five herbal medicine matrices were determined using CGIS in sextuplicate. The RSDs were found to meet the requirement set by the FGIS; the corresponding standard deviations (SD) are listed in Table S4. Consequently, the uncertainty range of the CGIS (Figure S5) (within which further confirmatory analysis was required) was established as 10 ± 2SD_max_ µg/kg, according to the DSTPSQATK Accuracy Rules of the FGIS.

**Figure 5:**
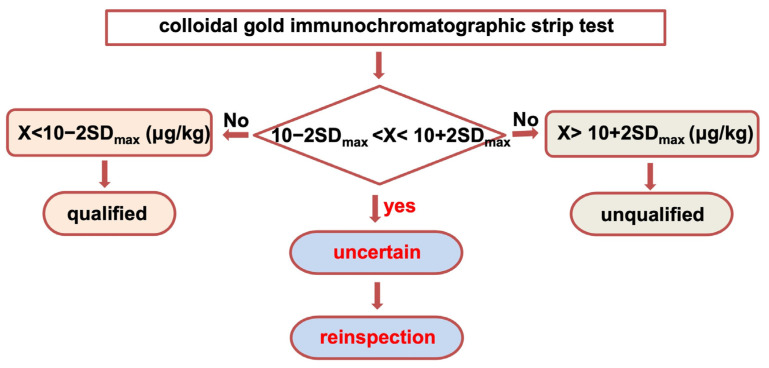
The flow chart for the process of judging the CGIS results. Sample results categorized as “uncertain” should be reinspected by HPLC-FLD or LC-MS/MS method for confirmation.

A process based on the FGIS guidelines was applied for the discrimination of the CGIS screening results; the flow chart for this process is illustrated in Figure 5. This process can be used to assess if samples meet the maximum allowable limits for total aflatoxins and to efficiently identify samples requiring further confirmatory testing in a laboratory, thus reducing the workload. The sample results were categorized as qualified, unqualified, or uncertain. Samples with values below 10 − 2SDmax µg/kg (8.6 μg/kg) were considered qualified (that is, compliant, below the maximum allowable limit), while those exceeding 10 + 2SDmax µg/kg (11.4 μg/kg) were deemed unqualified (that is, non-compliant, above the maximum allowable limit). Results within the range of uncertainty should be retested using a designated confirmatory reference method, such as HPLC-FLD or LC-MS/MS [27] to ensure accuracy.

### 2.6. Investigation of Real Samples by CGIS and HPLC-FLD

**Figure 6:**
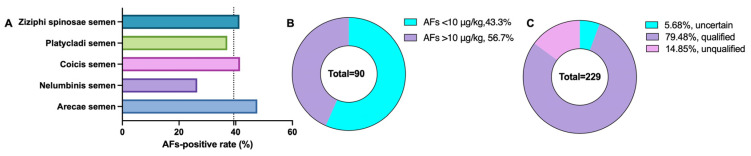
The distribution of the AFs-positive rate (**A**), AFs-positive samples at different levels (**B**), and sample results categorized as qualified, unqualified and uncertain (**C**).

Using the CGIS method, the total aflatoxin contamination in the authentic samples was judged following digitized detection. The results of the total aflatoxins, obtained using quantitative CGIS, are shown in Table S5. The total aflatoxin levels ranged from 2.6 to 39.2 μg/kg. The total aflatoxins-positive (AFs-positive) rate was 39.3% in 90 out of the 229 total samples, with positivity detected in 20 out of 40 *A. semen* samples, 9 out of 34 *N. semen* samples, 22 out of 53 *C. semen* samples, 20 out of 54 *P. semen* samples, and 19 out of 46 *Z. spinosae semen* samples. The highest AFs-positive rate was found in the *A. semen* samples (47.6%), which, along with the *C. semen* samples (41.5%) and the *Z. spinosae semen* samples (41.3%), had an above-average AFs-positive rate (39.3%; Table 2, Figure 6A). The *N. semen* samples had the lowest AFs-positive rate, thus demonstrating a lower AFs-exposure risk for *N. semen*. To make a further assessment of the AFs-exposure levels of the naturally occurring samples, the distribution of the AFs-positive samples and the AFs-positive rates at different levels were analyzed, as shown in Figure 6A,B. The Chinese Pharmacopoeia (2020 edition) prescribes a threshold of 10 µg/kg for total aflatoxins in herbal medicines. The AFs-positive level of 56.7% in 51 out of the 90 AFs-positive samples is lower than the maximum limit value in China (10 µg/kg). However, it is noteworthy that 43.3% of the 90 AFs-positive samples showed total aflatoxin levels of more than 10 µg/kg, exceeding the maximum limit value in China. These results indicate that the total aflatoxin contamination is a prevalent issue in herbal medicines. While most of the contamination levels remained within acceptable limits, some samples exhibited significantly high levels of aflatoxins, warranting further attention to ensure food safety and protect human health.

**Figure 7:**
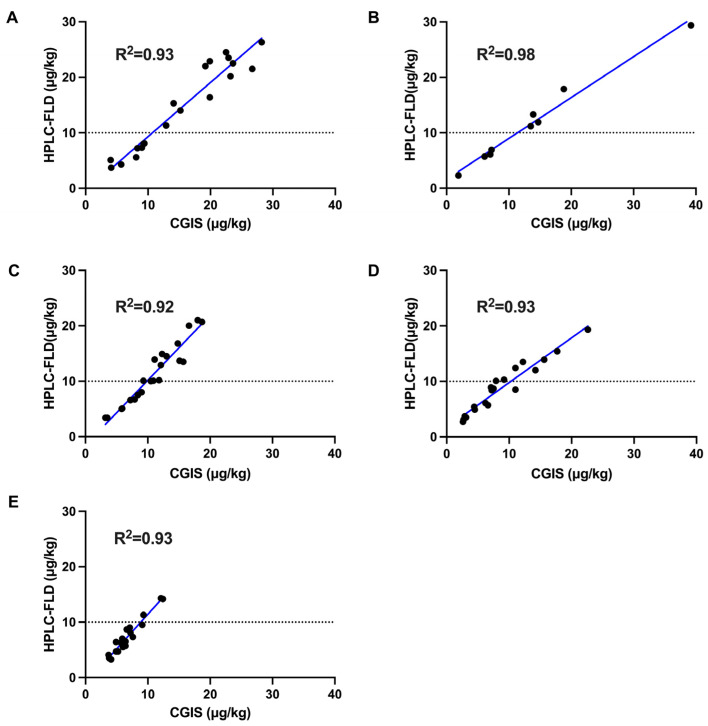
Correlation between CGIS and HPLC-FLD results of *A. semen* (**A**), *N. semen* (**B**), *C. semen* (**C**), *P. semen* (**D**), and *Z. spinosae semen* (**E**) samples.

The total aflatoxin contamination levels in the 229 samples were analyzed by the CGIS and HPLC-FLD method. The correlation between the CGIS and HPLC-FLD data is illustrated in Figure 7. The R^2^ in all 229 samples exceeded 0.92, and the agreements of the CGIS results with those from HPLC-FLD were 78.4–132.6% (*A. semen*), 82.6–133.0% (*N. semen*), 79.9–117.9% (*C. semen*), 78.1–119.0% (*P. semen*), and 76.1–123.0% (*Z. spinosae semen*), which further verifies the reliability and the accuracy of the proposed CGIS method. Furthermore, the proportion of CGIS results categorized as uncertain was 5.68% in all samples, among which the proportion of uncertainty in the *N. semen* samples was zero (Figure 6C). This represents a notable decrease in the number of samples requiring confirmatory testing and enhanced detection efficiency through the application of this novel CGIS method. Based on the data in our study, this process can save approximately 75% in costs and time.

## 3. Materials and Methods

### 3.1. Chemicals and Reagents

Afla-V strips and Afla-V ONE Diluent were purchased from Vicam (Waters, Milford, MA, USA). A CNtest total aflatoxins immunoaffinity column was obtained from Beijing Clovertech Ltd. (Beijing, China), and chromatographic grade methanol and acetonitrile were obtained from Fisher Chemical. Deionized water was prepared in a Milli-Q purification system (Merck KGaA, Darmstadt, Germany). AFB_1_, AFB_2_, AFG_1_, and AFG_2_, Ochratoxin A(OTA), O-methyl sterigmatocystin (o-m-ster), Deoxynivalenol (DON), Zearalenone (ZEN), and T-2 toxin (T-2) standards were obtained from SIGMA (Sigma-Aldrich, St. Louis, MO, USA), dissolved in acetonitrile, and stored at −20 °C.

### 3.2. CGIS Principle and Procedure

The whole workflow of the modified CGIS method is displayed in Scheme S1 (Supplementary Materials). The CGIS (Afla-V strip, Vicam Waters) comprised three pads (sample, conjugate, and absorbent pads) and a nitrocellulose membrane featuring two lines and colloidal gold-labeled monoclonal antibodies. A sample test mixture was placed on the sample pad and flowed laterally through the T (test) and C (control) lines before being immobilized by an immunoreaction [28,29]. In negative samples, bold red lines appear at both the T and C positions. However, in positive samples, the analytes in the sample compete against the immobilized test-line reagent and bind to the gold-labeled monoclonal antibodies, rendering a fainter T line, as illustrated in Figure S3. Therefore, a weaker T line intensity indicates a higher analyte concentration in the sample. The absence of a red line at the C position indicates an invalid test.

The analytical procedure entailed combining 200 µL of Afla-V ONE diluent and 100 µL of sample extract in a vial and gently shaking ten times. The test mixture (100 µL) was dropped vertically (~1 drop/second) onto the sample pad and the strip was allowed to develop on a flat surface for 5 min. During this incubation period, the analyte within the sample engaged in competitive binding with the antigen immobilized at the T line for the antibodies labeled with colloidal gold, resulting in a visibly lighter color at the T line. The determination of the detection result was based on measuring the alterations in absorbance at both the T line and the C line using a commercial Vertu TOUCH reader (Vicam Waters, USA). The ratio of the absorbance at the T line to that at the C line was utilized to generate calibration curves.

### 3.3. Evaluation of CGIS Sensitivity and Specificity

The sensitivity and specificity of the CGIS were evaluated based on its limit of detection (LOD) and cross-reactivity, respectively. To determine the LOD, gradually decreasing concentrations of aflatoxin standard were added to the blank matrices until there was no significant difference (*p* < 0.05) in signal compared to the blank matrix. The lowest concentration at which a significant difference in signal was evident was considered to be the LOD. The specificity was assessed using high concentrations (2 μg/mL) of other mycotoxins as the analytes, such as AFB_1_, AFB_2_, AFG_1_, AFG_2_ OTA, o-m-ster, DON, ZEN, and T-2.

### 3.4. Establishment of CGIS Calibration Curves

The use of naturally incurred (positive) samples to establish calibration curves can suffer from low reproducibility and incomplete concentration data, so the creation of matrix-matched calibration curves is preferable. Prior to building matrix-matched calibration curves, a specific contamination ratio model of AFB_1_, AFB_2_, AFG_1_, and AFG_2_ was established for each herbal medicine (*A. semen*, *N. semen*, *C. semen*, *P. semen*, and *Z. spinosae semen*). The aflatoxin contamination data from 1568 batches of these five herbal medicines was normalized, as the aflatoxin contamination levels were not normally distributed. The data were normalized and the result expressed as follows:

$$X_{normalization}=\frac{X_{i}-X_{min}}{X_{max}-X_{min}}$$

*X_min_* and *X_max_* were the minimum and maximum values from the contamination data of each herbal medicine, respectively. *X_i_* was the raw data. Briefly, the proportions of each aflatoxin contained in the samples were normalized, and the average of the normalized data for each aflatoxin was calculated. The ratio between these average values constituted the contamination ratio model.

Then, the mixed aflatoxin standard solutions based on the specific contamination ratio model were prepared for each herbal medicine. These solutions contained increasing concentrations of total aflatoxins, specifically 0, 2.5, 5, 7.5, 10, 15, 20, and 40 µg/kg, which were added into a blank matrix to generate calibration curves unique to each herbal medicine. The calibration curves were plotted using the ratios of the optical densities of the T and C lines against the logarithmic values of the calibrant concentrations (four-parameter logistic regression was applied to the data in GraphPad Prism 8.0). These matrix-matched calibrants were also subjected to HPLC-FLD analysis following purification by a total aflatoxins immunoaffinity column. The observed HPLC-FLD aflatoxin concentrations were used in the CGIS calibration curves to enhance the accuracy of the test results.

### 3.5. Detection of Spiked and Authentic Samples

The recovery tests of the spiked samples and the verification of the authentic samples by HPLC-FLD were used to evaluate the accuracy of the CGIS method for total aflatoxins detection. In total, 229 samples of *A. semen*, *N. semen*, *C. semen*, *P. semen*, and *Z. spinosae semen* were collected from herb markets in Shanghai (China), as detailed in Table S6. All the authentic samples were homogenized and stored at −20 °C before the detection procedure.

For the recovery test, the herbal medicine samples (3.0 g), which had been confirmed to be free of aflatoxin contamination, were completely crushed and the aflatoxin standard spiked at 5, 10 and 20 µg/kg. The spiked samples were mixed with 70% methanol (15 mL) and vortex mixed at 2200 rpm for 5 min, then centrifuged or allowed to stand for 5 min before the clear extract was recovered for analysis. Subsequently, the samples were detected using the above-mentioned CGIS method. The pretreatment and detection of the authentic samples were carried out in the same way as the above procedures.

To verify the reliability of the CGIS method, we used HPLC-FLD combined with aflatoxin immunoaffinity column purification to detect the total aflatoxins of all the authentic samples. The agreement was calculated to evaluate the correlation between the results of the CGIS and HPLC-FLD methods. The agreement was calculated as follows:

Agreement (%) = CGIS result/HPLC-FLD result × 100

The HPLC-FLD method was based on the method outlined in the Chinese Pharmacopoeia (2020 edition), with some modifications. In brief, 15 g of herbal medicine samples and 3 g of sodium chloride were put into tubes, followed by an addition of 75 mL of 70% methanol. The samples were then mixed at high speed (greater than 11,000 rpm/min) for 2 min, and centrifuged at 4000 rpm for 5 min. The supernatants (15 mL) were diluted with 35 mL of deionized water and centrifuged at 4000 rpm for 10 min. The diluted supernatants (20 mL) were subjected to aflatoxin immunoaffinity column purification, and the loaded column was washed with 20 mL of deionized water. Subsequently, water was squeezed out of the column and eluted with 1.5 mL of methanol. A total of 1.5 mL of the methanol eluate was diluted to 2.0 mL with deionized water, and this was used for the analyses. The samples were determined using a photochemical post-column derivatization reactor and a fluorescence detector (wavelengths: 360 nm excitation, 450 nm emission). A Shimadzu VP-ODS C18 column (4.6 × 250 mm, 5 µm) was used for chromatographic separation. The flow rate was 1.0 mL/min with an isocratic elution of 40% A (methanol), 18% B (acetonitrile), and 42% C (water) for 30 min.

Furthermore, to further evaluate the accuracy and precision of the CGIS method, three analysts independently extracted seven separate samples (*A. semen* samples were used as examples) and subjected them to analysis using the CGIS method, according to the DSTPSQATK Accuracy Rules established by the FGIS.

## 4. Conclusions

In this work, *A. semen*, *N. semen*, *C. semen*, *P. semen*, and *Z. spinosae semen* calibration curves were prepared by adding aflatoxin standards to a blank matrix, based on the ratios of AFB_1_, AFB_2_, AFG_1_, and AFG_2_ observed in contaminated herbal medicines recorded in an in-house database, which addressed the poor quantitative accuracy arising from the complexity of herbal medicine matrices. The quantitative range of the CGIS method was 2.5 to 40 μg/kg, and the LOD was 1 μg/kg. The accuracy of the CGIS method was validated by comparison with HPLC-FLD analysis. A discrimination process was applied to categorize the results of the CGIS method, reducing the need for confirmatory testing to less than 10% of the samples surveyed. This method holds the potential for allowing the rapid and accurate quantitative analysis of aflatoxins in herbal medicine samples. With the continuous improvement of aflatoxin contamination ratio models and the integration of rapid purification pretreatment methods, it is anticipated that this CGIS approach can be extended to more diverse types of herbal medicine matrices in the future, facilitating early risk warnings for aflatoxin contamination throughout the entire herbal medicine production process.

## Figures and Tables

**Table 1:** The ratio models of the four aflatoxins in the five herbal medicine matrices.

Herbal Medicine	Total Batch	Contamination Ratio of Aflatoxins				Conformity Rate (%)
		AFB_1_	AFB_2_	AFG_1_	AFG_2_	
*A. semen*	977	0.88	0.02	0.76	0.001	64.0
*N. semen*	102	0.95	0.11	0.002	0	88.9
*C. semen*	205	1.00	0.007	0	0	72.7
*P. semen*	144	0.94	0.07	0.006	0	83.3
*Z. spinosae semen*	140	0.96	0.06	0.009	0.003	95.5

**Table 2:** The investigation of the total aflatoxins in the authentic samples using the CGIS method.

Item	All	*A. semen*	*N. semen*	*C. semen*	*P. semen*	*Z.spinosae* *semen*
Total samples	229	42	34	53	54	46
AFs-positive samples	90	20	9	22	20	19
AFs-positive rate (%)	39.3	47.6	26.4	41.5	37.0	41.3
AFs-positive range (μg/kg)	2.6–39.2	4–28.2	3.8–39.2	3.2–18.7	2.6–22.6	3.7–12.4
Agreement (%)	-	78.4–132.6	82.6–133.0	79.9–117.9	78.1–119.0	76.1–123.0
Uncertain rate (%)	-	7.1	0	9.4	5.6	4.4

## Data Availability

The original contributions presented in the study are included in the article/Supplementary Materials; further inquiries can be directed to the corresponding authors.

## Supplementary Materials

The following supporting information can be downloaded at https://www.mdpi.com/article/10.3390/molecules29245827/s1. Scheme S1: Schematic illustration of determination of total aflatoxin in herbal medicine samples by CGIS method. Figure S1: Reactivity of the four types of aflatoxins with the same concentration (10 ng/mL) on the colloidal gold immunochromatographic scrips. Figure S2: The cross-reactivity tests of the CGIS for aflatoxin detection. Figure S3: The estimation plot between the negative sample and spiked sample (1 μg/kg) for *A. semen* (A), *N. semen*, (B), *C. semen* (C), *P. semen* (D), and *Z. spinosae semen* (E), respectively. If the 95% confidence interval includes 0, *p* > 0.05; if the 95% confidence interval does not include 0, *p* < 0.05. Figure S4: Different concentrations of aflatoxins (μg/kg) were determined using the CGIS method in *A. semen* (A), *N. semen* (B), *C. semen* (C), *P. semen* (D), and *Z. spinosae semen* (E), respectively. Figure S5: Schematic illustration of the uncertainty range in the process for the discrimination of the CGIS results. Table S1: The parameters and correlation coefficients of the four-parameter equation. Table S2: Recovery rate (RV) values for the total aflatoxins in the five herbal medicines. Table S3: Three analysts detected *A. semen* quality control samples (the total amount of aflatoxins was 8.0 μg/kg). Table S4: Detection of the total aflatoxins of authentic samples from five herbal medicines matrices using the CGIS method. Table S5: Comparison of the results of the CGIS and HPLC methods for the *A. semen*, *N. semen*, *C. semen*, *P. semen*, and *Z. spinosae semen* samples (n = 3). Table S6: The details of all the herbal medicine samples from major producing regions.
