# Supplementary material for: Detection of Total Aflatoxins in Herbal Medicines Based on Lateral Flow Assay with Contamination Ratio Model
Source: Molecules. 2024 Dec 10;29(24):5827. doi: 10.3390/molecules29245827 (PMC11728669; doi:10.3390/molecules29245827)
Supplement: Supplementary file 1 [file molecules-29-05827-s001.zip › molecules-3291790-supplementary.pdf]

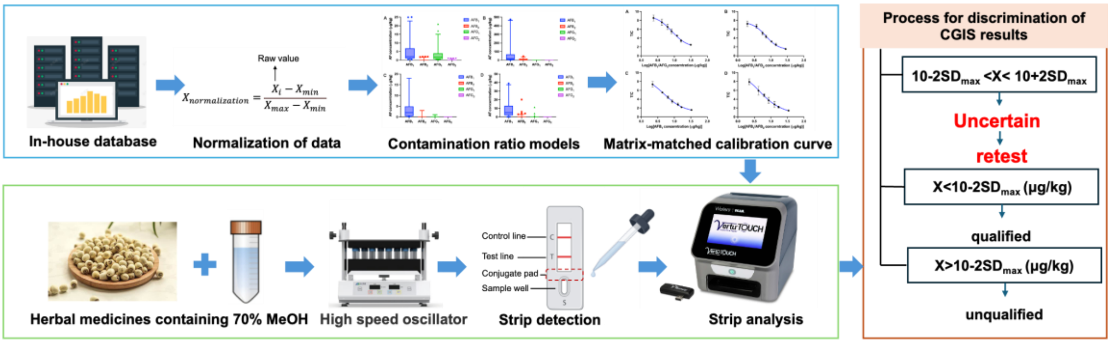


**Scheme S1**. Schematic illustration of determination of total aflatoxin in herbal medicine samples by CGIS method.

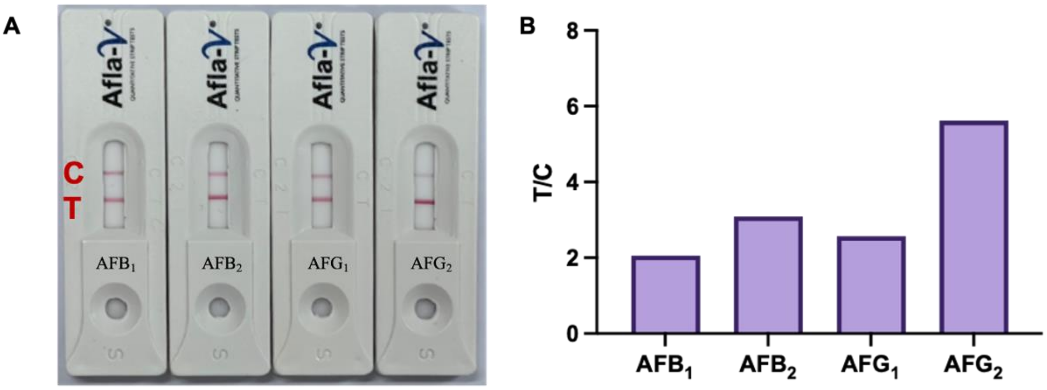


**Figure S1.** Reactivity of four types of aflatoxins with the same concentration (10 ng/mL) on the colloidal gold immunochromatographic scrips.

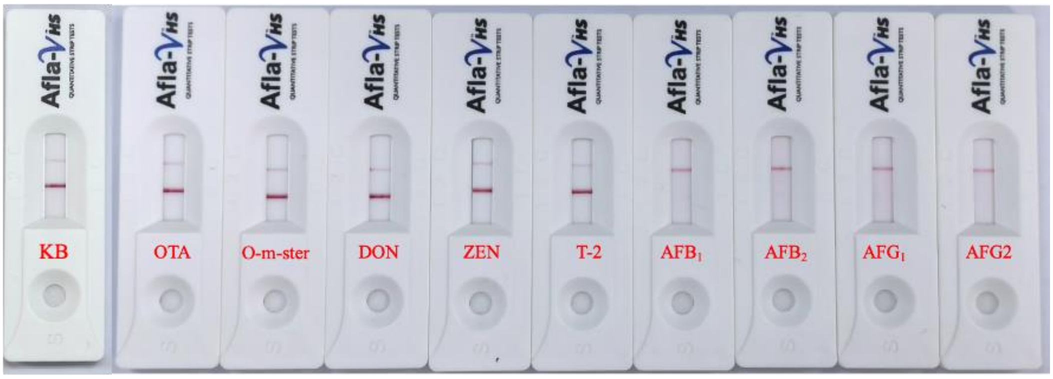


**Figure S2.** The cross-reactivity tests of the CGIS for aflatoxins detection.

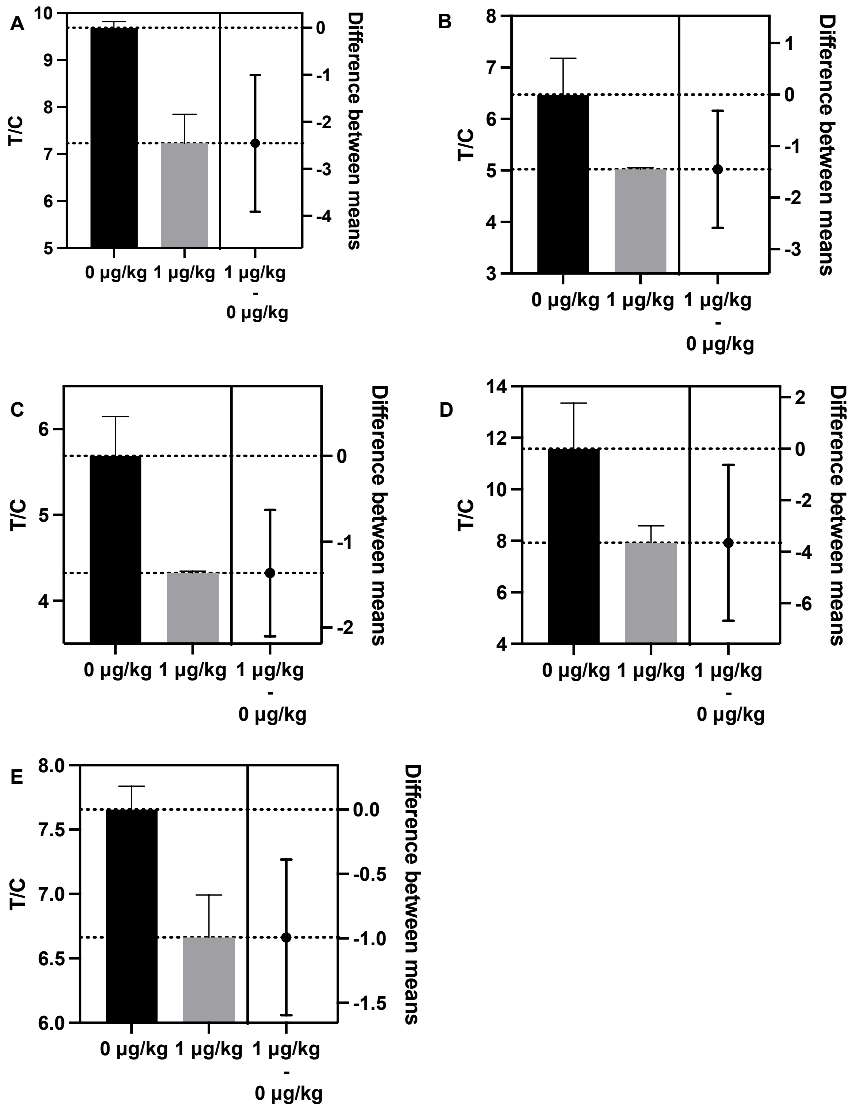


**Figure S3.** The estimation plot between negative sample and spiked sample (the total aflatoxins was 1 µg/kg) in AS (**A**), NS (**B**),CS(**C**), PS (**D**), and ZPS (**E**), respectively. If 95% confidence interval includes 0, $p>0.05$; If 95% confidence interval does not include 0, $p<0.05$.

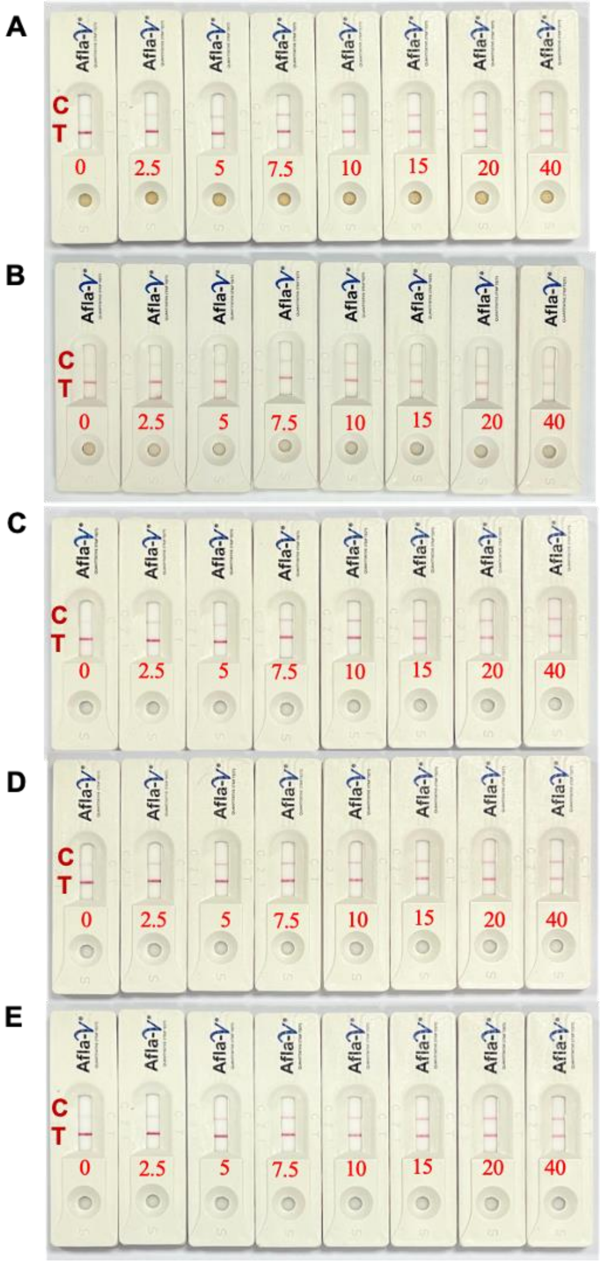


**Figure S4.** Different concentrations of aflatoxins (μg/kg) were determined using the CGIS method in AS(**A**), NS(**B**), CS(**C**), PS(**D**), and ZPS(**E**), respectively.

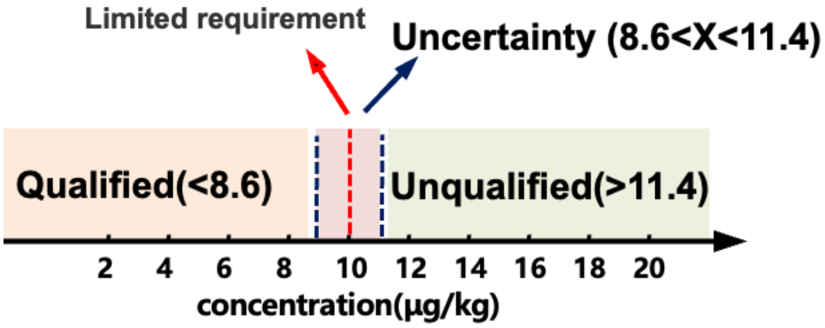


**Figure S5.** Schematic illustration of uncertainty range in the process for discrimination of CGIS results.

**Table S1.** The parameters and correlation coefficients of four parameters equation.

| Herbal medicine | $y = bottom + (top - bottom)/[1 + \left(\frac{IC_{50}}{x}\right)^{Hillslope}$ | | | | $R^2$ | LOD (μg/kg) |
|---|---|---|---|---|---|---|
| | bottom | top | $IC_{50}$ | hillslope | | |
| AS | 1.86 | 9.15 | 8.48 | -1.86 | 0.96 | 1.0 |
| NS | 1.30 | 7.70 | 6.99 | -2.19 | 0.97 | 1.0 |
| CS | 1.02 | 9.52 | 4.73 | -1.39 | 0.99 | 1.0 |
| PS | -0.21 | 8.60 | 0.87 | -2.67 | 0.95 | 1.0 |
| ZPS | 1.05 | 8.84 | 3.27 | -1.23 | 0.99 | 1.0 |
| Solvent standard | -1.784 | 3.515 | 1.629 | -1.925 | 0.99 | - |

**Table S2.** Recovery rate (RV) values for total aflatoxins in five herbal medicines.

| Herbal medicine | spiked level (μg/kg) | measured level (μg/kg) | RV (%) | Average RV (%) | RSD (%) |
|---|---|---|---|---|---|
| AS | 5 | 4.5 | 89.0 | 96.7 | 8.8 |
| | | 4.8 | 95.4 | | |
| | | 5.3 | 105.8 | | |
| | 10 | 7.1 | 71.3 | 86.0 | 14.9 |
| | | 9.2 | 92.1 | | |
| | | 9.5 | 94.6 | | |
| | 20 | 18.0 | 90.0 | 84.5 | 11.1 |
| | | 14.7 | 73.7 | | |
| | | 18.0 | 89.9 | | |
| NS | 5 | 3.5 | 70.4 | 71.4 | 2.7 |
| | | 3.7 | 73.6 | | |
| | | 3.5 | 70.2 | | |
| | 10 | 7.0 | 70.1 | 75.2 | 10.0 |
| | | 7.2 | 71.7 | | |
| | | 8.4 | 83.8 | | |
| | 20 | 12.2 | 60.9 | 67.8 | 11.9 |
| | | 13.2 | 65.9 | | |
| | | 15.3 | 76.7 | | |
| CS | 5 | 4.5 | 89.6 | 90.0 | 1.6 |
| | | 4.6 | 91.6 | | |
| | | 4.4 | 88.8 | | |
| | 10 | 10.4 | 104.1 | 89.8 | 13.9 |
| | | 8.4 | 84.0 | | |
| | | 8.1 | 81.3 | | |
| | 20 | 16.2 | 80.9 | 81.5 | 6.5 |
| | | 17.4 | 87.1 | | |
| | | 15.3 | 76.6 | | |
| PS | 5 | 4.1 | 81.8 | 98.6 | 16.9 |
| | | 4.9 | 98.8 | | |

| | | | | | |
|---|---|---|---|---|---|
| | | 5.8 | 115.2 | | |
| | | 7.4 | 74.3 | | |
| | 10 | 7.1 | 71.2 | 90.2 | 12.5 |
| | | 9.0 | 89.5 | | |
| | | 18.8 | 93.9 | | |
| | 20 | 15.0 | 74.8 | 81.5 | 13.1 |
| | | 15.2 | 76.0 | | |
| | | 3.7 | 73.0 | | |
| | 5 | 3.9 | 77.6 | 72.2 | 8.1 |
| | | 3.3 | 66.0 | | |
| | | 6.3 | 63.3 | | |
| ZPS | 10 | 6.6 | 65.8 | 65.1 | 2.4 |
| | | 6.6 | 66.2 | | |
| | | 15.4 | 77.2 | | |
| | 20 | 13.0 | 64.9 | 70.5 | 8.8 |
| | | 13.9 | 69.4 | | |

**Table S3.** Three analysts detected AS quality control samples (the total of aflatoxin was 8.0 μg/kg).

| NO. | CGIS method (μg/kg) |
|---|---|
| | 9.6 |
| | 8.9 |
| | 9.9 |
| analysts 1 | 8.7 |
| | 8.5 |
| | 10.2 |
| | 8.1 |
| | 7.7 |
| | 7.6 |
| | 9.0 |
| analysts 2 | 9.9 |
| | 7.7 |
| | 8.8 |
| | 7.4 |
| | 8.9 |
| | 8.3 |
| | 7.7 |
| analysts 3 | 9.9 |
| | 9.6 |
| | 9.0 |
| | 9.3 |
| Average | 8.8 |
| RSD (%) | 9.6 |

**Table S4.** Detection of total aflatoxins of authentic samples from five herbal medicines matrices using the CGIS method.

| NO. | AS | NS | CS | PS | ZPS |
|---|---|---|---|---|---|
| | measured level (μg/kg) | measured level (μg/kg) | measured level (μg/kg) | measured level (μg/kg) | measured level (μg/kg) |
| 1. | 9.6 | 8.5 | 7.7 | 8.3 | 7.6 |
| 2. | 8.9 | 7.2 | 7.6 | 8.0 | 7.6 |
| 3. | 9.9 | 6.6 | 6.4 | 8.1 | 6.9 |
| 4. | 8.7 | 7.7 | 8.2 | 6.8 | 6.3 |
| 5. | 9.3 | 7.9 | 7.3 | 7.9 | 7.0 |
| 6. | 10.2 | 6.9 | 8.4 | 7.7 | 7.7 |
| Average | 9.4 | 7.5 | 7.6 | 7.8 | 7.2 |
| SD | 0.6 | 0.7 | 0.7 | 0.5 | 0.5 |
| RSD (%) | 6.1 | 9.4 | 9.4 | 6.8 | 7.6 |
| HPLC (μg/kg) | 8.0 | 6.9 | 7.5 | 10.1 | 8.1 |
| Agreement (%) | 117.5 | 108.7 | 101.3 | 77.2 | 88.8 |

**Table S5.** Comparison the results of CGIS method and HPLC in AS, NS, CS, PS, and ZPS samples (*n*=3).

| Herbal medicine | Batch | HPLC-FLD (μg/kg) | CGIS (μg/kg) | Agreement (%) | Results |
|---|---|---|---|---|---|
| AS | AS-1 | 5.1 | 4.0 | 78.4 | qualified |
| | AS-2 | 14.0 | 15.2 | 108.6 | unqualified |
| | AS-3 | 4.3 | 5.2 | 120.9 | qualified |
| | AS-4 | 7.2 | 8.3 | 115.3 | qualified |
| | AS-5 | 8.1 | 9.4 | 116.0 | uncertain |
| | AS-6 | 16.4 | 19.9 | 121.3 | unqualified |
| | AS-7 | 23.5 | 22.9 | 97.4 | unqualified |
| | AS-8 | 22.9 | 19.9 | 86.9 | unqualified |
| | AS-9 | 22.0 | 19.2 | 87.3 | unqualified |
| | AS-10 | 8.0 | 9.3 | 116.3 | uncertain |
| | AS-11 | 7.3 | 9.0 | 123.3 | uncertain |
| | AS-12 | 11.3 | 12.9 | 114.2 | unqualified |
| | AS-13 | 21.5 | 26.7 | 124.2 | unqualified |
| | AS-14 | 22.5 | 23.6 | 104.9 | unqualified |
| | AS-15 | 24.5 | 22.5 | 91.8 | unqualified |
| | AS-16 | 15.3 | 14.1 | 92.2 | unqualified |
| | AS-17 | 26.3 | 28.2 | 107.2 | unqualified |
| | AS-18 | 20.2 | 23.2 | 114.9 | unqualified |
| | AS-19 | 3.7 | 4.1 | 110.8 | qualified |
| | AS-20 | 5.6 | 6.8 | 121.4 | qualified |
| | AS-21~32 | 0.2~2.5 | >LOD | / | qualified |
| | AS-33~42 | nd | / | / | qualified |
| NS | NS-1 | 5.7 | 6.1 | 107.0 | qualified |
| | NS-2 | 6.9 | 7.2 | 104.3 | qualified |

| | | | | | |
|---|---|---|---|---|---|
| | NS-3 | 6.1 | 7.0 | 114.8 | qualified |
| | NS-4 | 13.3 | 13.9 | 104.5 | unqualified |
| | NS-5 | 11.9 | 14.7 | 123.4 | unqualified |
| | NS-6 | 4.6 | 3.8 | 82.6 | qualified |
| | NS-7 | 11.2 | 13.5 | 120.0 | unqualified |
| | NS-8 | 17.9 | 18.8 | 105.0 | unqualified |
| | NS-9 | 29.4 | 39.2 | 133.0 | unqualified |
| | NS-10~23 | 0.9~2.5 | >LOD | / | qualified |
| | NS-24~34 | nd | / | / | qualified |
| CS | CS-1 | 3.4 | 3.2 | 94.1 | qualified |
| | CS-2 | 10.1 | 9.3 | 92.1 | uncertain |
| | CS-3 | 3.4 | 3.5 | 102.9 | qualified |
| | CS-4 | 14.9 | 12.3 | 82.6 | unqualified |
| | CS-5 | 5.1 | 5.9 | 115.7 | qualified |
| | CS-6 | 10.1 | 10.9 | 107.9 | uncertain |
| | CS-7 | 20.7 | 18.7 | 90.5 | unqualified |
| | CS-8 | 6.6 | 7.2 | 109.1 | qualified |
| | CS-9 | 6.7 | 7.9 | 117.9 | qualified |
| | CS-10 | 8.0 | 9.0 | 112.5 | uncertain |
| | CS-11 | 7.5 | 8.4 | 112.0 | unqualified |
| | CS-12 | 13.5 | 15.7 | 116.3 | unqualified |
| | CS-13 | 13.7 | 15.1 | 110.2 | unqualified |
| | CS-14 | 5.0 | 5.8 | 116.0 | qualified |
| | CS-15 | 13.9 | 11.1 | 79.9 | uncertain |
| | CS-16 | 16.8 | 14.8 | 88.1 | unqualified |
| | CS-17 | 21.0 | 18.0 | 85.8 | unqualified |
| | CS-18 | 10.0 | 10.5 | 105.0 | uncertain |
| | CS-19 | 12.9 | 12.1 | 93.8 | unqualified |
| | CS-20 | 14.5 | 13.0 | 89.7 | unqualified |
| | CS-21 | 20.0 | 16.6 | 83.0 | unqualified |
| | CS-22 | 10.2 | 11.8 | 115.7 | unqualified |
| | CS-23~41 | 0.9~2.5 | >LOD | / | qualified |
| | CS-42~53 | nd | / | / | qualified |
| PS | PS-1 | 13.9 | 15.6 | 112.3 | unqualified |
| | PS-2 | 12.0 | 14.2 | 119.0 | unqualified |
| | PS-3 | 15.4 | 17.7 | 115.0 | unqualified |
| | PS-4 | 19.3 | 22.6 | 116.9 | unqualified |
| | PS-5 | 13.5 | 12.2 | 90.8 | unqualified |
| | PS-6 | 8.5 | 11.0 | 128.7 | uncertain |
| | PS-7 | 6.1 | 6.2 | 101.1 | qualified |
| | PS-8 | 4.9 | 4.5 | 90.8 | qualified |
| | PS-9 | 5.4 | 4.4 | 82.1 | qualified |
| | PS-10 | 8.7 | 7.5 | 85.8 | qualified |
| | PS-11 | 5.7 | 6.6 | 114.6 | qualified |

| | | | | | |
|---|---|---|---|---|---|
| | PS-12 | 3.7 | 2.9 | 80.5 | qualified |
| | PS-13 | 8.9 | 7.1 | 79.3 | qualified |
| | PS-14 | 2.7 | 2.6 | 94.8 | qualified |
| | PS-15 | 12.4 | 11.0 | 89.2 | uncertain |
| | PS-16 | 3.1 | 2.7 | 88.4 | qualified |
| | PS-17 | 3.5 | 3.1 | 90.1 | qualified |
| | PS-18 | 10.3 | 9.2 | 89.0 | uncertain |
| | PS-19 | 8.4 | 7.2 | 86.1 | qualified |
| | PS-20 | 10.1 | 7.9 | 78.1 | qualified |
| | PS-23~40 | 1.2~2.5 | >LOD | / | qualified |
| | PS-41~54 | nd | / | / | qualified |
| | ZSS-1 | 4.1 | 3.7 | 91.0 | qualified |
| | ZSS-2 | 8.7 | 6.6 | 76.1 | qualified |
| | ZSS-3 | 6.4 | 4.9 | 76.4 | qualified |
| | ZSS-4 | 3.3 | 4.1 | 123.0 | qualified |
| | ZSS-5 | 6.3 | 5.7 | 89.8 | qualified |
| | ZSS-6 | 3.5 | 3.8 | 107.1 | qualified |
| | ZSS-7 | 7.3 | 7.6 | 104.7 | qualified |
| | ZSS-8 | 4.7 | 4.9 | 104.4 | qualified |
| | ZSS-9 | 5.5 | 6.0 | 107.5 | qualified |
| | ZSS-10 | 14.3 | 12.1 | 84.5 | unqualified |
| ZPS | ZSS-11 | 6.5 | 6.4 | 98.6 | qualified |
| | ZSS-12 | 7.0 | 5.9 | 85.3 | qualified |
| | ZSS-13 | 5.7 | 6.4 | 112.7 | qualified |
| | ZSS-14 | 9.0 | 7.1 | 79.0 | qualified |
| | ZSS-15 | 8.1 | 7.2 | 89.2 | qualified |
| | ZSS-16 | 9.5 | 9.1 | 95.9 | uncertain |
| | ZSS-17 | 11.3 | 9.3 | 82.1 | uncertain |
| | ZSS-18 | 4.7 | 5.2 | 110.4 | qualified |
| | ZSS-19 | 14.2 | 12.4 | 87.2 | unqualified |
| | ZSS-20~28 | 0.4~2.5 | >LOD | / | qualified |
| | ZSS-29~46 | nd | / | / | qualified |

**Table S6.** The details of all herbal medicine samples from major producing regions.

| Herbal Medicine | Batch | origin | Collection Time |
|---|---|---|---|
| | AS-1 | Indonesia | Dec-2021 |
| | AS-2 | Indonesia | Dec-2021 |
| | AS-3 | Indonesia | Dec-2021 |
| | AS-4 | Indonesia | Dec-2021 |
| AS | AS-5 | Indonesia | Dec-2021 |
| | AS-6 | Indonesia | Dec-2021 |
| | AS-7 | Indonesia | Dec-2021 |
| | AS-8 | Indonesia | Dec-2021 |
| | AS-9 | Indonesia | Dec-2021 |

| | | | |
|---|---|---|---|
| | AS-10 | Indonesia | Dec-2021 |
| | AS-11 | Indonesia | Dec-2021 |
| | AS-12 | Indonesia | Dec-2021 |
| | AS-13 | Shanghai, China | Dec-2021 |
| | AS-14 | Shanghai, China | Dec-2021 |
| | AS-15 | Shanghai, China | Dec-2021 |
| | AS-16 | Shanghai, China | Dec-2021 |
| | AS-17 | Shanghai, China | Dec-2021 |
| | AS-18 | Shanghai, China | Dec-2021 |
| | AS-19 | Shanghai, China | Dec-2021 |
| | AS-20 | Shanghai, China | Dec-2021 |
| | AS-21 | Indonesia | Dec-2021 |
| | AS-22 | Indonesia | Dec-2021 |
| | AS-23 | Indonesia | Dec-2021 |
| | AS-24 | Indonesia | Dec-2021 |
| | AS-25 | Indonesia | Dec-2021 |
| | AS-26 | Indonesia | Dec-2021 |
| | AS-27 | Indonesia | Dec-2021 |
| | AS-28 | Indonesia | Dec-2021 |
| | AS-29 | Indonesia | Dec-2021 |
| | AS-30 | Indonesia | Dec-2021 |
| | AS-31 | Indonesia | Dec-2021 |
| | AS-32 | Indonesia | Dec-2021 |
| | AS-33 | Indonesia | Dec-2021 |
| | AS-34 | Indonesia | Dec-2021 |
| | AS-35 | Indonesia | Dec-2021 |
| | AS-36 | Indonesia | Dec-2021 |
| | AS-37 | Indonesia | Dec-2021 |
| | AS-38 | Indonesia | Dec-2021 |
| | AS-39 | Indonesia | Dec-2021 |
| | AS-40 | Shanghai, China | Dec-2021 |
| | AS-41 | Shanghai, China | Dec-2021 |
| | AS-42 | Shanghai, China | Dec-2021 |
| | NS-1 | Shanghai, China | Sep-2021 |
| | NS-2 | Shanghai, China | Sep-2021 |
| | NS-3 | Hunan, China | Sep-2021 |
| | NS-4 | Hunan, China | Sep-2021 |
| | NS-5 | Hunan, China | Sep-2021 |
| NS | NS-6 | Hunan, China | Sep-2021 |
| | NS-7 | Hunan, China | Sep-2021 |
| | NS-8 | Hunan, China | Sep-2021 |
| | NS-9 | Hunan, China | Sep-2021 |
| | NS-10 | Hunan, China | Sep-2021 |
| | NS-11 | Hunan, China | Sep-2021 |

| | | | |
|---|---|---|---|
| | NS-12 | Hunan, China | Sep-2021 |
| | NS-13 | Hunan, China | Sep-2021 |
| | NS-14 | Hunan, China | Sep-2021 |
| | NS-15 | Hunan, China | Sep-2021 |
| | NS-16 | Hunan, China | Sep-2021 |
| | NS-17 | Hunan, China | Sep-2021 |
| | NS-18 | Hunan, China | Sep-2021 |
| | NS-19 | Hunan, China | Sep-2021 |
| | NS-20 | Hunan, China | Sep-2021 |
| | NS-21 | Hunan, China | Sep-2021 |
| | NS-22 | Hunan, China | Sep-2021 |
| | NS-23 | Hunan, China | Sep-2021 |
| | NS-24 | Hunan, China | Sep-2021 |
| | NS-25 | Hunan, China | Sep-2021 |
| | NS-26 | Hunan, China | Sep-2021 |
| | NS-27 | Hunan, China | Sep-2021 |
| | NS-28 | Hunan, China | Sep-2021 |
| | NS-29 | Hunan, China | Sep-2021 |
| | NS-30 | Shanghai, China | Sep-2021 |
| | NS-31 | Shanghai, China | Sep-2021 |
| | NS-32 | Shanghai, China | Sep-2021 |
| | NS-33 | Shanghai, China | Sep-2021 |
| | NS-34 | Shanghai, China | Sep-2021 |
| CS | CS-1 | Shanghai, China | Dec-2021 |
| | CS-2 | Shanghai, China | Dec-2021 |
| | CS-3 | Shanghai, China | Dec-2021 |
| | CS-4 | Shanghai, China | Dec-2021 |
| | CS-5 | Shanghai, China | Dec-2021 |
| | CS-6 | Hunan, China | Dec-2021 |
| | CS-7 | Hunan, China | Dec-2021 |
| | CS-8 | Hunan, China | Dec-2021 |
| | CS-9 | Hunan, China | Dec-2021 |
| | CS-10 | Hunan, China | Dec-2021 |
| | CS-11 | Hunan, China | Dec-2021 |
| | CS-12 | Hunan, China | Dec-2021 |
| | CS-13 | Hunan, China | Dec-2021 |
| | CS-14 | Shanghai, China | Dec-2021 |
| | CS-15 | Shanghai, China | Dec-2021 |
| | CS-16 | Shanghai, China | Dec-2021 |
| | CS-17 | Shanghai, China | Dec-2021 |
| | CS-18 | Shanghai, China | Dec-2021 |
| | CS-19 | Shanghai, China | Dec-2021 |
| | CS-20 | Shanghai, China | Dec-2021 |
| | CS-21 | Shanghai, China | Dec-2021 |

| | | | |
|---|---|---|---|
| | CS-22 | Shanghai, China | Dec-2021 |
| | CS-23 | Shanghai, China | Dec-2021 |
| | CS-24 | Shanghai, China | Dec-2021 |
| | CS-25 | Shanghai, China | Dec-2021 |
| | CS-26 | Shanghai, China | Dec-2021 |
| | CS-27 | Shanghai, China | Dec-2021 |
| | CS-28 | Shanghai, China | Dec-2021 |
| | CS-29 | Shanghai, China | Dec-2021 |
| | CS-30 | Shanghai, China | Dec-2021 |
| | CS-31 | Shanghai, China | Dec-2021 |
| | CS-32 | Shanghai, China | Dec-2021 |
| | CS-33 | Shanghai, China | Dec-2021 |
| | CS-34 | Shanghai, China | Dec-2021 |
| | CS-35 | Shanghai, China | Dec-2021 |
| | CS-36 | Shanghai, China | Dec-2021 |
| | CS-37 | Shanghai, China | Dec-2021 |
| | CS-38 | Shanghai, China | Dec-2021 |
| | CS-39 | Shanghai, China | Dec-2021 |
| | CS-40 | Shanghai, China | Dec-2021 |
| | CS-41 | Shanghai, China | Dec-2021 |
| | CS-42 | Shanghai, China | Dec-2021 |
| | CS-43 | Shanghai, China | Dec-2021 |
| | CS-44 | Shanghai, China | Dec-2021 |
| | CS-45 | Shanghai, China | Dec-2021 |
| | CS-46 | Shanghai, China | Dec-2021 |
| | CS-47 | Shanghai, China | Dec-2021 |
| | CS-48 | Shanghai, China | Dec-2021 |
| | CS-49 | Shanghai, China | Dec-2021 |
| | CS-50 | Shanghai, China | Dec-2021 |
| | CS-851 | Shanghai, China | Dec-2021 |
| | CS-52 | Shanghai, China | Dec-2021 |
| | CS-53 | Shanghai, China | Dec-2021 |
| PS | PS-1 | Henan, China | Dec-2021 |
| | PS-2 | Henan, China | Dec-2021 |
| | PS-3 | Henan, China | Dec-2021 |
| | PS-4 | Henan, China | Dec-2021 |
| | PS-5 | Henan, China | Dec-2021 |
| | PS-6 | Henan, China | Dec-2021 |
| | PS-7 | Henan, China | Dec-2021 |
| | PS-8 | Shandong, China | Dec-2021 |
| | PS-9 | Shandong, China | Dec-2021 |
| | PS-10 | Shandong, China | Dec-2021 |
| | PS-11 | Shandong, China | Dec-2021 |
| | PS-12 | Shandong, China | Dec-2021 |

| | | | |
|---|---|---|---|
| | PS-13 | Shandong, China | Dec-2021 |
| | PS-14 | Shandong, China | Dec-2021 |
| | PS-15 | Shandong, China | Dec-2021 |
| | PS-16 | Shandong, China | Dec-2021 |
| | PS-17 | Hebei, China | Dec-2021 |
| | PS-18 | Hebei, China | Dec-2021 |
| | PS-19 | Hebei, China | Dec-2021 |
| | PS-20 | Hebei, China | Dec-2021 |
| | PS-21 | Hebei, China | Dec-2021 |
| | PS-22 | Hebei, China | Dec-2021 |
| | PS-23 | Hebei, China | Dec-2021 |
| | PS-24 | Hebei, China | Dec-2021 |
| | PS-25 | Hebei, China | Dec-2021 |
| | PS-26 | Hebei, China | Dec-2021 |
| | PS-27 | Hebei, China | Dec-2021 |
| | PS-28 | Hebei, China | Dec-2021 |
| | PS-29 | Hebei, China | Dec-2021 |
| | PS-30 | Hebei, China | Dec-2021 |
| | PS-31 | Henan, China | Dec-2021 |
| | PS-32 | Henan, China | Dec-2021 |
| | PS-33 | Henan, China | Dec-2021 |
| | PS-34 | Henan, China | Dec-2021 |
| | PS-35 | Henan, China | Dec-2021 |
| | PS-36 | Henan, China | Dec-2021 |
| | PS-37 | Henan, China | Dec-2021 |
| | PS-38 | Henan, China | Dec-2021 |
| | PS-39 | Henan, China | Dec-2021 |
| | PS-40 | Henan, China | Dec-2021 |
| | PS-41 | Henan, China | Dec-2021 |
| | PS-42 | Henan, China | Dec-2021 |
| | PS-43 | Henan, China | Dec-2021 |
| | PS-44 | Henan, China | Dec-2021 |
| | PS-45 | Henan, China | Dec-2021 |
| | PS-46 | Shandong, China | Dec-2021 |
| | PS-47 | Shandong, China | Dec-2021 |
| | PS-48 | Shandong, China | Dec-2021 |
| | PS-49 | Shandong, China | Dec-2021 |
| | PS-50 | Shandong, China | Dec-2021 |
| | PS-51 | Shandong, China | Dec-2021 |
| | PS-52 | Shandong, China | Dec-2021 |
| | PS-53 | Shandong, China | Dec-2021 |
| | PS-54 | Shandong, China | Dec-2021 |
| ZPS | ZPS-1 | Shandong, China | Sep-2021 |
| | ZPS-2 | Shandong, China | Sep-2021 |

| | | | |
|---|---|---|---|
| | ZPS-3 | Shandong, China | Sep-2021 |
| | ZPS-4 | Shandong, China | Sep-2021 |
| | ZPS-5 | Shandong, China | Sep-2021 |
| | ZPS-6 | Shandong, China | Sep-2021 |
| | ZPS-7 | Shandong, China | Sep-2021 |
| | ZPS-8 | Shanghai, China | Sep-2021 |
| | ZPS-9 | Shanghai, China | Sep-2021 |
| | ZPS-10 | Shanghai, China | Sep-2021 |
| | ZPS-11 | Shanghai, China | Sep-2021 |
| | ZPS-12 | Shanghai, China | Sep-2021 |
| | ZPS-13 | Shanghai, China | Sep-2021 |
| | ZPS-14 | Shanghai, China | Sep-2021 |
| | ZPS-15 | Shanghai, China | Sep-2021 |
| | ZPS-16 | Shanghai, China | Sep-2021 |
| | ZPS-17 | Shanghai, China | Sep-2021 |
| | ZPS-18 | Hebei, China | Sep-2021 |
| | ZPS-19 | Hebei, China | Sep-2021 |
| | ZPS-20 | Hebei, China | Sep-2021 |
| | ZPS-21 | Hebei, China | Sep-2021 |
| | ZPS-22 | Hebei, China | Sep-2021 |
| | ZPS-23 | Hebei, China | Sep-2021 |
| | ZPS-24 | Hebei, China | Sep-2021 |
| | ZPS-25 | Hebei, China | Sep-2021 |
| | ZPS-26 | Hebei, China | Sep-2021 |
| | ZPS-27 | Hebei, China | Sep-2021 |
| | ZPS-28 | Hebei, China | Sep-2021 |
| | ZPS-29 | Hebei, China | Sep-2021 |
| | ZPS-30 | Hebei, China | Sep-2021 |
| | ZPS-31 | Hebei, China | Sep-2021 |
| | ZPS-32 | Hebei, China | Sep-2021 |
| | ZPS-33 | Hebei, China | Sep-2021 |
| | ZPS-34 | Hebei, China | Sep-2021 |
| | ZPS-35 | Hebei, China | Sep-2021 |
| | ZPS-36 | Hebei, China | Sep-2021 |
| | ZPS-37 | Hebei, China | Sep-2021 |
| | ZPS-38 | Hebei, China | Sep-2021 |
| | ZPS-39 | Hebei, China | Sep-2021 |
| | ZPS-40 | Hebei, China | Sep-2021 |
| | ZPS-41 | Hebei, China | Sep-2021 |
| | ZPS-42 | Hebei, China | Sep-2021 |
| | ZPS-43 | Hebei, China | Sep-2021 |
| | ZPS-44 | Hebei, China | Sep-2021 |
| | ZPS-45 | Hebei, China | Sep-2021 |
| | ZPS-46 | Hebei, China | Sep-2021 |
